# Supplementary material for: Content-rich biological network constructed by mining PubMed abstracts
Source: BMC Bioinformatics. 2004 Oct 8;5:147. doi: 10.1186/1471-2105-5-147 (PMC528731; doi:10.1186/1471-2105-5-147)
Supplement: Additional File 2 — The original results of the above study (non-essential files are deleted to keep the file size under the limit set by BMC bioinformatics). [file 1471-2105-5-147-S2.bz2 › chilibotAdditionalFile2/dip20/240ID9063449E787/html/MMP1_TIMP1.html]

 


 **MMP1** and **TIMP1** 
  
Found 2107 abstracts in PubMed, retrieved 20.  
 

 What does Google say? 
 PDF only 
| .edu only 

---

**Interactive relationship** (e.g. stimulation, inhibition, etc)

**Inhibitory relationship**- We show that TIMP 1  [ **TIMP1** ]  can be transformed into an active inhibitor against MT1 MMP  [ **MMP1** ]  by the mutation of a single residue, namely, threonine 98 to leucine T98L.  Ref: 12869573 J Biol ChemJ Biol Chem,
- The minidegradome constituted of two matrix metalloproteinases MMP  [ **MMP1** ] , i.e. MMP  [ **MMP1** ]  2 and MT1 MMP  [ **MMP1** ] , associated with tissue inhibitor of metalloprotease 2 TIMP  [ **TIMP1** ]  2 and integrin alpha v beta 3 or CD 44, is mainly involved in such invasive program.  Ref: 12888258 Biomed Pharmacother, 2003
**Stimulatory relationship**- The enzymatic activity of MT1 MMP  [ **MMP1** ]  is regulated by endogenous inhibitors, the tissue inhibitor of metalloproteinases  [ **TIMP1** ]  TIMP  [ **TIMP1** ] .  Ref: 12869573 J Biol ChemJ Biol Chem, 2003
**Neutral relationship**

**Non-interactive relationship** (e.g. studied together, co-existance, homology, etc.)

- Low dose estrogen could decrease the ratio of MMP 1  [ **MMP1** ]  TIMP 1  [ **TIMP1** ]  and MMP  [ **MMP1** ]  3 TIMP 1  [ **TIMP1** ] , which could even be decreased by progestin.  Ref: 12887766 Zhonghua Yi Xue Za Zhi, 2003
- Our findings establish an unexpected, MMP  [ **MMP1** ]  independent mechanism for TIMP  [ **TIMP1** ]  2 inhibition of endothelial cell proliferation in vitro and reveal an important component of the antiangiogenic effect of TIMP2 in vivo.  Ref: 12887919 Cell, 2003
- , MMP  [ **MMP1** ]  and 9 activities were upregulated, and TIMP  [ **TIMP1** ]  2 was expressed.  Ref: 12873454 Exp Eye Res, 2003
- There were no changes in the expression or activity of MMP  [ **MMP1** ]  inhibitors, TIMP  [ **TIMP1** ] , and 4.  Ref: 12875773 J Am Coll Cardiol, 2003
- Among the patients with strong grades 2 and 3 MMP  [ **MMP1** ]  9 staining positivity for TIMP 1  [ **TIMP1** ]  indicated a trend toward a more favorable prognosis.  Ref: 12890147 Eur J Haematol, 2003
- Gene expressions of type I and II collagen, metalloproteinase 1 and 3 MMP 1  [ **MMP1** ]  and 3, and tissue inhibitor of metalloproteinase 1  [ **TIMP1** ]  TIMP 1  [ **TIMP1** ]  as well as the IL 1beta induced gene expressions of MMP 1  [ **MMP1** ]  and 3 were analyzed by reverse transcription polymerase chain reaction RT PCR.  Ref: 12880581 Osteoarthritis CartilageOsteoarthritis Cartilage, 2003
- The purpose of this study was to characterize effects of cytokines, a phorbol ester, and prostanoids on the expression of MMP  [ **MMP1** ] , 3, and 9 and tissue inhibitors of metalloproteinases TIMP 1  [ **TIMP1** ]  and 2 in cultured human TM cells.  Ref: 12882798 Invest Ophthalmol Vis Sci, 2003
- Extracellular matrix molecule degradation related genes, including matrix metalloproteinase MMP  [ **MMP1** ]  10, MMP  [ **MMP1** ]  13, MMP  [ **MMP1** ]  15, tissue inhibitor of metalloproteinase TIMP  [ **TIMP1** ]  2, TIMP  [ **TIMP1** ]  3, plasminogen and plasminogen activator, were also highly expressed in the pre eclamptic placenta, compared to the normal placenta.  Ref: 12866918 Br J Biomed Sci, 2003
- Precise mRNA levels of  **TIMP1** ,  **MMP1** , and beta spectrin were determined using quantitative real time reverse transcription polymerase chain reaction RT PCR of myocardial samples from 27 patients with deteriorating heart failure who underwent LVAD implantation, from 17 patients with stable ESF who underwent elective heart transplantation, and from 28 donor organs with good hemodynamic function.Gridded macro arrays analysis of pooled failing heart samples determined that  **TIMP1**  mRNA was the most readily detectable TIMP  [ **TIMP1** ]  in failing myocardium.  Ref: 12873541 J Heart Lung Transplant, 2003
- Finally, we observed a decrease of MMP 1  [ **MMP1** ] , MMP  [ **MMP1** ]  3, MMP  [ **MMP1** ]  9, and MT1 MMP  [ **MMP1** ] , both at the mRNA and at the protein levels, in E cadherin positive clones whereas no changes in MMP  [ **MMP1** ]  2, TIMP 1  [ **TIMP1** ] , or TIMP  [ **TIMP1** ]  2 were observed when compared with control clones.  Ref: 12875984 Am J Pathol, 2003
- Serum levels of IL 6, interstitial collagenase  [ **MMP1** ]  MMP 1  [ **MMP1** ] , stromelysin 1 MMP  [ **MMP1** ]  3, gelatinase B MMP  [ **MMP1** ]  9, TIMP 1  [ **TIMP1** ]  and TIMP  [ **TIMP1** ]  2 were assessed with an ELISA technique in 30 RA patients.  Ref: 12879774 Pol Arch Med Wewn, 2003
- The majority of these processes requires a balanced equilibrium between the activity of matrix metalloproteinases MMP  [ **MMP1** ]  and their natural tissue inhibitors TIMP  [ **TIMP1** ] .  Ref: 12879657 Ceska Gynekol, 2003
- The protein synthesis of MMP 1  [ **MMP1** ]  and 3 and TIMP 1  [ **TIMP1** ]  was determined by Western blotting.RESULTS The treatment of chondrocytes with DHEA did not affect chondrocyte proliferation or GAG synthesis up to 100μM of concentration.  Ref: 12880581 Osteoarthritis CartilageOsteoarthritis Cartilage, 2003
- We suggest that assessing the serum IL 6, MMP 1  [ **MMP1** ] , MMP  [ **MMP1** ]  3, MMP  [ **MMP1** ]  9 and TIMP 1  [ **TIMP1** ]  levels may be helpful in the prediction of the RA activity.  Ref: 12879774 Pol Arch Med Wewn, 2003
- Both  **TIMP1**  and  **MMP1**  showed positive correlation with each other and with previously determined levels of mRNA for both interleukin 1beta IL 1beta and IL 6 in this patient series when considering all patients individually, but neither correlated with tumor necrosis factor alpha.Patients with deteriorating heart failure have increased expression of  **TIMP1**  and  **MMP1**  mRNA.  Ref: 12873541 J Heart Lung Transplant, 2003
- Unveiling the surface epitopes that render tissue inhibitor of metalloproteinase TIMP 1  [ **TIMP1** ]  inactive against membrane type 1 matrix metalloproteinase MT1 MMP  [ **MMP1** ] .  Ref: 12869573 J Biol ChemJ Biol Chem, 2003
- Whilst TIMP  [ **TIMP1** ] , and 4 are potent inhibitors against MT1 MMP  [ **MMP1** ] , TIMP 1  [ **TIMP1** ]  displays negligible inhibitory activity against the enzyme.  Ref: 12869573 J Biol ChemJ Biol Chem, 2003
- MMP  [ **MMP1** ]  2, MMP  [ **MMP1** ]  9, MMP  [ **MMP1** ]  8, TIMP 1  [ **TIMP1** ]  and TIMP  [ **TIMP1** ]  2 were detected by immunohistochemistry and the Western blot technique.  Ref: 12873454 Exp Eye Res, 2003
- The invasive potential of Ad p16 transduced cells was evaluated using a Matrigel invasion assay, and trimolecular complex MMP  [ **MMP1** ]  2 MT1 MMP  [ **MMP1** ]  TIMP  [ **TIMP1** ]  2 synthesis was proven by zymography and Western blotting.  Ref: 12883667 Oncol RepOncol Rep, 2003
- Organ cultures were performed using explants from the skin of three patients from the same family with recessive dystrophic epidermolysis bullosa to reveal and quantify the expression of MMP 1  [ **MMP1** ]  collagenase 1, MMP  [ **MMP1** ]  2 and MMP  [ **MMP1** ]  9 gelatinases A and B, MMP  [ **MMP1** ]  3 stromelysin 1, TIMP 1  [ **TIMP1** ] , and TIMP  [ **TIMP1** ]  2, and to compare the results with those obtained with two human control skins, with the same experimental conditions.  Ref: 12880418 J Invest Dermatol, 2003
- Our findings also provide a potential mechanistic explanation for MMP  [ **MMP1** ]  TIMP  [ **TIMP1** ]  selectivity.  Ref: 12869573 J Biol ChemJ Biol Chem, 2003
- OBJECTIVES The present study was carried out to clarify the role of matrix metalloproteinase 2 and 9 MMP  [ **MMP1** ]  2 and MMP  [ **MMP1** ]  9, tissue inhibitor of metalloproteinase 1  [ **TIMP1** ]  TIMP 1  [ **TIMP1** ]  and the extent of neovascularization in the clinicopathologic behavior of non Hodgkin s lymphomas.  Ref: 12890147 Eur J Haematol, 2003
- Matrix metalloproteinases MMP  [ **MMP1** ]  2 and 9 and tissue inhibitors of matrix metalloproteinases TIMP 1  [ **TIMP1** ]  and 2 during the course of experimental necrotizing herpetic keratitis.  Ref: 12873454 Exp Eye Res, 2003
- We demonstrate here that TIMP  [ **TIMP1** ]  2 abrogates angiogenic factor induced endothelial cell proliferation in vitro and angiogenesis in vivo independent of MMP  [ **MMP1** ]  inhibition.  Ref: 12887919 Cell, 2003
- We hypothesized that matrix metalloproteinases MMPs and their endogenous inhibitors, the tissue inhibitors of MMPs TIMPs, would be implicated in the mechanisms that underlie deteriorating heart failure.Gridded macro array filters were used to provide a broad overview of MMP  [ **MMP1** ]  and TIMP  [ **TIMP1** ]  mRNA expression in heart failure.  Ref: 12873541 J Heart Lung Transplant, 2003
- Reverse transcription polymerase chain reaction were used to detect the mRNA expression of MMP 1  [ **MMP1** ] , MMP  [ **MMP1** ]  3, TIMP 1  [ **TIMP1** ]  and IL 1beta in the OA synovia to determine the effect of estrogen and progestin.  Ref: 12887766 Zhonghua Yi Xue Za Zhi, 2003
- 0.001, and the MMP  [ **MMP1** ]  inhibitor TIMP  [ **TIMP1** ]  2 P = 0.025.  Ref: 12893184 Gynecol Oncol, 2003
- Platelet derived growth factor BB was generally ineffective in modulating MMP  [ **MMP1** ]  and TIMP  [ **TIMP1** ]  levels.  Ref: 12882798 Invest Ophthalmol Vis Sci, 2003
- So we suspected that the phenotypic variability for the same mutation could be linked to other genetic or environmental factors, as a particular balance between MMP  [ **MMP1** ]  and TIMP  [ **TIMP1** ] .  Ref: 12880418 J Invest Dermatol, 2003
- Our results showed a significant decrease of collagenases MMP  [ **MMP1** ]  8 and MMP  [ **MMP1** ]  13 with an increase of TIMP 1  [ **TIMP1** ]  and transforming growth factor beta.  Ref: 12882763 Am J Physiol Lung Cell Mol PhysiolAm J Physiol Lung Cell Mol Physiol, 2003
- OBJECTIVE To observe the effect of different dose of estrogen or estrogen plus progestin on the mRNA expression of MMP 1  [ **MMP1** ] , MMP  [ **MMP1** ]  3, TIMP 1  [ **TIMP1** ]  and IL 1beta in synovia of female New Zealand White rabbit OA model.  Ref: 12887766 Zhonghua Yi Xue Za Zhi, 2003
- Increased expression of extracellular matrix regulators  **TIMP1**  and  **MMP1**  in deteriorating heart failure.  Ref: 12873541 J Heart Lung Transplant, 2003
- The expression of collagenases MMP  [ **MMP1** ]  and 13 and tissue inhibitor of metalloproteinases  [ **TIMP1** ]  TIMP 1  [ **TIMP1** ]  was altered in OA cartilage, but no difference was detected between lesion and distal sites in the same jointi.e. no correlation was found between senescent cells and proteinase inhibitor expression.  Ref: 12882354 Aging Cell, 2002
- However, we were unable to demonstrate any differences in the constitutive productions and secretions of MMP  [ **MMP1** ]  2, MT1 MMP  [ **MMP1** ] , and TIMP  [ **TIMP1** ]  2, among the mock treated, Ad lacZ transduced, and Ad p16 transduced cells.  Ref: 12883667 Oncol RepOncol Rep, 2002
- Furthermore for this patient we evidenced higher amounts of MMP 1  [ **MMP1** ]  and also a lower TIMP 1  [ **TIMP1** ]  amount in his unlesioned and lesioned skin compared with the other two affected patients and with healthy control donors.  Ref: 12880418 J Invest Dermatol, 2003
- However, the mRNAs coding for urokinase type PA uPA, membrane type 1 MMP  [ **MMP1** ]  MT1 MMP  [ **MMP1** ] , and tissue inhibitor of metalloproteinases  [ **TIMP1** ]  type 3 TIMP  [ **TIMP1** ]  3 were constantly expressed in the mouse CL throughout its whole life span.  Ref: 12890575 Mol Cell Endocrinol, 2003
- METHODS Paraffin embedded histologic sections from 57 patients with aggressive non Hodgkin s lymphomas were stained with MMP  [ **MMP1** ]  2, MMP  [ **MMP1** ]  9, TIMP 1  [ **TIMP1** ] , and factor VIII antibodies to correlate the expression of these markers to the clinical disease characteristics.  Ref: 12890147 Eur J Haematol, 2003
- So we can suspect that recessive dystrophic epidermolysis bullosa phenotypic variability could be related to patients collagenase activity heterogeneity, linked to imbalance between MMP 1  [ **MMP1** ]  and TIMP 1  [ **TIMP1** ] .  Ref: 12880418 J Invest Dermatol, 2003
- Collagenase and stromelysin activities have been found to be overexpressed in skin cultures of some recessive dystrophic epidermolysis bullosa patients, and tissue destruction in the disease process might result from an imbalance of metalloproteinases MMP  [ **MMP1** ]  over tissueinhibitor of metalloproteinases TIMP  [ **TIMP1** ] .  Ref: 12880418 J Invest Dermatol, 2003
- In this study we examined the sequential expression of several matrix metalloproteinases MMP  [ **MMP1** ] , tissue inhibitors of metalloproteinases TIMP  [ **TIMP1** ] , and growth factors as well as the presence of apoptosis in a model of pulmonary fibrosis induced in rats with paraquat and hyperoxia.  Ref: 12882763 Am J Physiol Lung Cell Mol PhysiolAm J Physiol Lung Cell Mol Physiol, 2003
- Here, we identify the surface epitopes that render TIMP 1  [ **TIMP1** ]  inactive against MT1 MMP  [ **MMP1** ] .  Ref: 12869573 J Biol ChemJ Biol Chem, 2003
- Gelatinases MMP  [ **MMP1** ]  2 and MMP  [ **MMP1** ]  9, TIMP 1  [ **TIMP1** ]  expression and the extent of neovascularization in aggressive non Hodgkin s lymphomas.  Ref: 12890147 Eur J Haematol, 2003
